# The Effect of Feeding Sequence on the Structure and Properties of the Ethylene/1-Octene Copolymer in the Semi-Continuous Polymerization Reaction System

**DOI:** 10.3390/polym16040526

**Published:** 2024-02-15

**Authors:** Qiqi He, Ruijun Zhang, Yuexin Hu, Junhua Li, Hongbo Yu, Yong Zheng, Jianhua Qian

**Affiliations:** 1 College of Chemistry and Chemical Engineering, China University of Petroleum (East China), Qingdao 266555, China; qqhe65878@gmail.com (Q.H.); zhangruijun523@gmail.com (R.Z.); 2 College of Petrochemical Engineering, Liaoning Petrochemical University, Fushun 113001, China; yxhu1981@163.com (Y.H.); lijunhua0521@163.com (J.L.); 3 PetroChina Fushun Petrochemical, Fushun 113001, China; yhbfssh@petrochina.com.cn; 4 College of Materials and Chemical Engineering, China Three Gorges University, Yichang 443002, China

**Keywords:** ethylene-1-octene copolymer, feeding sequence, chain microstructure, copolymer property

## Abstract

The performance of ethylene/1-octene copolymer primarily depends on the microstructure of the polymer chain. This study employed a new method to control the inter-distribution of hexyl chain branches directly on the backbone of the ethylene/1-octene copolymer. Three ethylene/1-octene copolymers with different inter-distributions of hexyl chain branches were synthesized using [Me_2_Si(C_5_Me_4_) (N*^t^*Bu)] TiCl_2_ (Ti–CGC) by different feeding sequences in the semi-continuous polymerization reaction system. The three copolymers were named according to the feeding sequence of the materials: ethylene/1-octene/Ti–CGC (EOC), 1-octene/Ti–CGC/ethylene (OCE), and ethylene/Ti–CGC/1-octene (ECO), respectively. The structure and properties of the copolymers were characterized using HT-GPC, ^13^C-NMR, DSC, WAXD, DMA, MI, and Uniaxial Tension Test. The results showed that the feeding sequence greatly affected the comonomer distribution of the molecular chains, molecular weight, molecular weight distribution, and chemical composition of the copolymers, consequently influencing their thermal performance and mechanical properties. Thus, it is probable that one could obtain an ethylene/1-octene copolymer with designed properties by controlling the feeding sequence during the ethylene/1-octene semi-continuous copolymerization process.

## 1. Introduction

Polyolefin elastomer (POE) is a type of ethylene/α-olefin copolymer. POE comprises ethylene as the main polymer unit and α-olefin as the comonomer, with the content of α-olefin greater than 20 wt%. Due to the high comonomer content, the polymer chain consists of a crystalline resin and amorphous rubber phases. As a result, this material exhibits both the high elasticity of rubber and the plasticity of thermoplastic resins, making it easy to process and mold [1,2]. POE is widely used in photovoltaics, automobiles, buildings, cables, etc. Especially in photovoltaics, POE films [3] exhibit superior aging resistance, low water vapor permeability, and anti-PID (Potential-Induced Degradation) properties. With the continuous advancement of photovoltaics, the prospects of POE films are full of imagination.

Ethylene/1-octene copolymer is an excellent POE due to its long branch. Ethylene/1-octene copolymer was initially synthesized using Ziegler–Natta catalysts [4,5,6]. However, these copolymers have a low 1-octene incorporation, resulting in poor elastic properties. DOW developed “Engage” products using single-site metallocene catalysts [7,8]. These copolymers exhibit a narrow molecular weight distribution and excellent material processing properties. Current research on POE is focused on developing novel copolymer catalysts [9,10,11,12,13,14,15,16,17,18,19,20,21], but there are still significant challenges in controlling copolymer chain microstructures. The performance of POE materials primarily depends on the microstructure of polymer segments. Therefore, the design and analysis of segment microstructure is crucial for developing new properties in POE [22,23,24,25,26,27,28,29,30]. Mitsui Chemicals in Japan has developed a benzoxazine (FI) ligand catalyst, which exhibits excellent ethylene/1-octene live copolymerization when all the hydrogen atoms on the benzene ring are replaced with fluorine [31]. This catalyst allows for precise control over the microstructure of the polymer chains, enabling the production of novel polyolefin products. However, it has low catalytic efficiency and poor thermal stability, making it unsuitable for industrial high-temperature solution polymerization methods. Zhang [27] carried out the synthesis of a comb-like ethylene/1-octene copolymer using a tandem catalyst approach. The chain branches are composed of crystalline polyethylene prepared with a FI–Zr catalyst, and the ethylene/1-octene/PE macromonomer copolymer was prepared with a Ti–CGC catalyst. The copolymer exhibits high melting points and low glass transition temperatures. DOW developed OBC in chain shuttling polymerization [32]. OBC consists of alternating crystalline and amorphous segments with high melting and low glass transition temperatures.

**Scheme 1:**
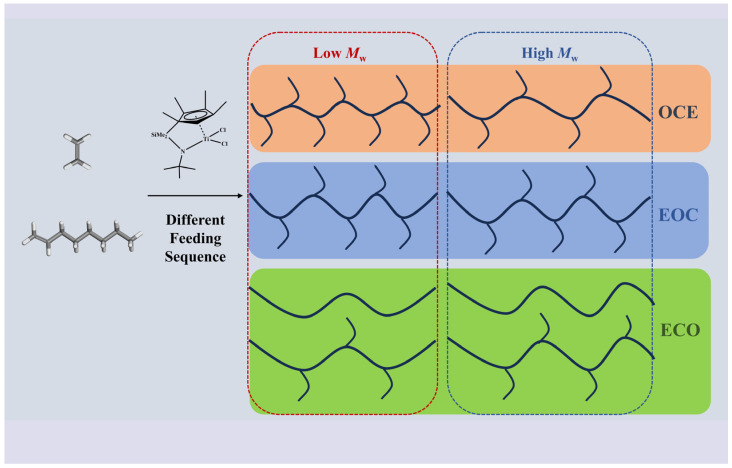
Scheme diagram of synthetic ethylene/1-octene copolymer with different inter-distributions of hexyl chain branches.

The chain structure design method of a tandem catalyst and chain shuttle polymerization is very complex. Chi [33] prepared poly (ester imide)s with different chain structures by changing monomers’ feeding sequence; the synthesized products have different liquid-crystalline properties, a fiber-forming ability, and other properties. Inspired by this method, one aims to synthesize ethylene/1-octene copolymers with a different inter-distribution of hexyl chain branches by adjusting the feeding sequence of ethylene, 1-octene, and the catalyst. Scheme 1 shows the scheme diagram of the synthetic ethylene/1-octene copolymer with different inter-distributions of hexyl chain branches. The influence of ethylene, 1-octene, and the catalyst feeding sequence on the copolymer structure and properties was investigated.

## 2. Materials and Methods

### 2.1. Materials

Methylaluminoxane (MAO, 10 wt% in toluene) from Sigma-Aldrich (St. Louis, MO, USA) was used as received. Ethylene was procured from Shenyang Shuntai Special Gas Co., Ltd. (Shenyang City, Liaoning Province, China), underwent purification via molecular sieves and copper oxide prior to utilization. 4A Molecular sieves and copper oxide were acquired from Shanghai Aladdin Biochemical Technology Co., Ltd. (Fengxian District, Shanghai, China). Cyclohexane and 1-octene were obtained from Shanghai Maclin Biochemical Technology Co., Ltd. (Pudong, Shanghai, China) and were dried through molecular sieves; [Me_2_Si(C_5_Me_4_) (N*^t^*Bu)] TiCl_2_ (Ti–CGC) was supplied by Beijing Research Institute of Chemical Engineering (Beijing, China). All air sensitive compounds were taken from the glove box.

### 2.2. Characterization and Performance Testing

#### 2.2.1. High-Temperature Gel Permeation Chromatography (HT-GPC)

The molecular weight (*M*_w_ and *M*_n_) and molecular weight distribution (*M*_w_/*M*_n_) were determined using HT-GPC (Polymer Char, Valencia City, Spain). The GPC system was equipped with infrared and differential viscosity detectors. The solvent used 1,2,4-trichlorobenzene, and the flow rate was set at 1.0 mL/min. The dissolution time was set to 90 min to ensure complete dissolution. A narrow molecular weight distribution polystyrene standard was used in the universal calibration.

#### 2.2.2. ^13^C-Nuclear Magnetic Resonance (^13^C-NMR)

The copolymer chemical composition was analyzed using ^13^C-NMR, which was performed on a JEOL JNM-ECZL400S NMR spectrometer(JEOL, Tokyo, Japan) at 125 °C [34]. The polymer was dissolved in a 10% deuterated ortho-dichlorobenzene (o-DCB) solution at 150 °C. The solution was dissolved for 3 h to ensure uniformity. The instrument parameters were optimized with a 90° pulse angle, reverse gated proton decoupling (decoupled spectrum without NOE), a 1.3 S acquisition time, an 8 S pulse delay (*R*_d_ > 5*T*_1_), and an 8000 Hz spectral width. The NMR analysis involved an average of at least 5000 scans. The most substantial peak was identified at 30 ppm as a reference.

#### 2.2.3. Differential Scanning Calorimetry (DSC)

The melting point (*T*_m_) and crystallinity (*X*_c_) of the copolymer were determined by TA Q20 DSC (TA Instruments, New Castle, DE, USA). The nitrogen flow rate was set at 50 mL/min. The polymer sample (8.0–10.0 mg) was heated to 200 °C at 10 °C/min, kept for 5 min to eliminate thermal history, and then cooled to 0 °C at 10 °C/min, kept for 5 min, and heated to 200 °C at 10 °C/min. The polymer melting point was obtained from the second melting curve, and the crystallinity was calculated through the melting peak area:

(1) $$X_{c}=\Delta H/\Delta H_{0}$$

where ∆*H*_0_ = 293 J/g.

The copolymer’s Successive Self-Nucleation Annealing (SSA) was determined by DSC (Q20, TA). Heat treatment was performed on samples (8–10 mg) according to the following temperature control program: (1) heat to 200 °C and maintain a constant temperature for 5 min to eliminate heat history; (2) cool to 20 °C at 10 °C/min; (3) heat to the first self-nucleation temperature (*T*_s_) at 10 °C/min; (4) complete the first self-nucleation and annealing process at the first *T*_s_ for 5 min; (5) cool to 0 °C at 10 °C/min; (6) repeat the above process, and complete continuous self-nucleation annealing at a lower *T*_s_ each time. The first *T*_s_ of EOC and OCE copolymers was set to 121 °C, the first *T*_s_ of the ECO copolymers was set to 126 °C, and the grading window was set to 5 °C. Each annealing time was set to 5 min, and all heating and cooling rates were set to 10 °C/min.

#### 2.2.4. Wide Angle X-ray Diffraction (WAXD)

The sample analyzed by TD-3500 WAXD (Dandong Tongda Science & Technology Co., Ltd, Dandong City, Liaoning Province, China) is a circular plate with a thickness of 1.5 mm and a diameter of 20 mm. The scan range was 5–30°, the scan increment was 0.02°, the scanning voltage was 40 kV, and the scanning current was 30 mA.

#### 2.2.5. Melting Index (MI)

The samples were compressed using a flat vulcanizing machine, and the upper and lower mold temperatures were set at 190 °C. The samples were compressed multiple times to remove air and ensure sample consolidation. After cooling the samples to room temperature, they were cut into small squares of approximately 3 mm for further testing.

The melt flow rate (*MFR*) of the copolymer was determined using a MI40 instrument (Goettfert, Germany) according to the Chinese National Standard [35]. The melt flow rate instrument was maintained at 190 °C for at least 15 min. The copolymer sample (2.5–3.0 g) was added. The sample melted for 5 min. Then, under a load of 2.16 kg, the sample was extruded to form a filament, and the *MFR* was measured.

#### 2.2.6. Dynamic Mechanical Analysis (DMA)

Dynamic Thermal-Mechanical properties were performed using the DMA (DMA Q800 analyzer, TA instruments, USA) in tension mode. The samples used were long strips with dimensions of 1 × 4 mm. The following testing conditions were applied: an amplitude of 20 um, a scanning starting temperature of −80 °C, a termination temperature of 40 °C, and a heating rate of 3 °C/min.

#### 2.2.7. Uniaxial Tension Test

The uniaxial tensile properties of the material were tested using an electronic universal testing machine CMT 4503 (MTS, Eden Prairie, MN, USA). The test was conducted at room temperature with a tensile speed of 100 mm/min and a load cell capacity of 10 kN. The sample dimensions were as follows: a width of 4 mm and a thickness of 1 mm. Each test was performed on three parallel samples for accurate results.

### 2.3. Polymerization Experiment

The solution copolymerization of ethylene/1-octene occurred in a 5 L stainless steel reactor. Cyclohexane and 1-octene were pumped into the reactor using a diaphragm pump, and ethylene was pressurized using a compressor before being introduced into the reactor. Three types of copolymers were named according to the feeding sequence: ethylene/1-octene/Ti–CGC (EOC), 1-octene/Ti–CGC/ethylene (OCE), and ethylene/Ti–CGC/1-octene (ECO) copolymers, respectively.

#### 2.3.1. Preparation before Reaction

Cyclohexane (3 L) was added to a stainless-steel reactor (5 L). The temperature was set at 120 °C, and the stirring speed was set to 600 rpm. The reactor was then washed for 1 h. Afterward, the reactor was dried at 120 °C for 1 h. The reactor was evacuated for 1 h and flushed several times with nitrogen.

#### 2.3.2. Catalyst Preparation

The entire catalyst preparation process was carried out inside the glovebox. Firstly, two conical flasks (100 mL), labeled A and B, were dried at 120 °C for 30 min. Subsequently, cyclohexane (20 mL) was added to Flask A. Next, Ti–CGC (28 mg) was introduced, and the solution was thoroughly mixed using magnetic stirring for 2 min. In Flask B, MAO (6 mL) solution was added, followed by the prepared catalyst solution (5 mL) from Flask A. Flask B was extensively mixed using a magnetic stirrer for 5 min. Afterward, the catalyst solution from Flask B was drawn into a syringe (20 mL), and the needle was sealed with a rubber stopper. Simultaneously, triethylaluminum (3 mL) (reaction system impurity removal) was aspirated into a syringe (10 mL) for later use.

#### 2.3.3. Preparation of EOC Copolymer

The reactor was charged with cyclohexane (1 L) and stirred at a rate of 600 rpm. The temperature was then increased to 120 °C, and the ethylene pressure was increased to 3 MPa. Sequentially, 1-octene (300 mL) and triethylaluminum (3 mL) were added. Under a nitrogen atmosphere, a pre-configured catalyst solution was introduced into the reactor using a long needle (20 cm). An additional cyclohexane (1 L) was supplemented. Ethylene was continuously supplied through a control system to maintain a constant reaction pressure (3 MPa). The timer was started, and the reaction proceeded for 15 min. Subsequently, the ethylene feed valve was closed, and the pressure relief valve was opened. The reaction mixture was swiftly transferred into a glass cylinder containing an ethanol solution with HCl (10 wt%). The copolymer was naturally dried (24 h), followed by a vacuum drying process at 60 °C (24 h).

#### 2.3.4. Preparation of OCE Copolymer

The reactor was charged with cyclohexane (1 L) and stirred at a rate of 600 rpm. The temperature was then increased to 120 °C. Subsequently, 1-octene (300 mL) and triethylaluminum (3 mL) were added in the given sequence. Under a nitrogen atmosphere, a pre-prepared catalyst solution was carefully introduced into the reactor using a long needle (20 cm). An additional cyclohexane (1 L) was added. The ethylene pressure was gradually increased to 3 MPa within 5 min, and a timer was started to monitor the reaction time. The ethylene supply was maintained through a controlled system to ensure a constant reaction pressure of 3 MPa. After 15 min, the ethylene feed valve was closed, and the pressure relief valve was opened. The reaction mixture was swiftly transferred into a glass cylinder containing an ethanol solution with HCl (10 wt%). The resulting copolymer was naturally dried (24 h), followed by a vacuum drying process at 60 °C (24 h).

#### 2.3.5. Preparation of ECO Copolymer

The reactor was charged with cyclohexane (1 L), stirred at a rate of 600 rpm, and heated to 120 °C. The ethylene pressure was increased to 3 MPa, followed by the addition of triethylaluminum (3 mL). Under a nitrogen atmosphere, a pre-prepared catalyst solution was added using a long needle (20 cm) through a controlled system. An additional cyclohexane (1 L) was added. Finally, 1-octene (300 mL) was added within 5 min, starting timing after the completion of 1-octene feeding. During the reaction, ethylene was continuously supplied through the control system to maintain a constant reaction pressure (3 MPa). After 15 min, the ethylene feed valve was closed, and the pressure relief valve was opened. The reaction mixture was quickly transferred to a glass cylinder containing an ethanol solution of HCl (10 wt%). The copolymer was naturally dried (24 h) and then subjected to vacuum drying at 60 °C (24 h).

## 3. Results and Discussion

The microstructure of the copolymer is determined by the content and sequence distribution of ethylene and 1-octene in the copolymer. In homogeneous solution polymerization, the ethylene/1-octene copolymerization equation is represented by Equation (2). By selecting different monomer ratios under constant temperature, pressure, and catalyst conditions, copolymerization can be carried out in various ways to obtain a wide range of copolymers with diverse properties. This improves various properties in the ethylene/1-octene copolymer, such as mechanical strength and elasticity.

(2) $$F_{e}=\frac{r_{e}f_{e}+f_{e}f_{o}}{r_{e}f_{e}^{2}+2f_{e}f_{o}+r_{o}f_{o}^{2}}$$

where *F*_e_ stands for the molar fraction of ethylene in the copolymer, *f*_e_ and *f*_o_ stand for the mole fractions of monomers ethylene and 1-octene, and *r*_e_ and *r*_o_ stand for the monomer reactivity ratio.

In ethylene/1-octene copolymerization, changing the feeding sequence will cause changes in the initial concentration of ethylene and 1-octene, resulting in copolymers with different 1-octene compositional distributions. Based on the ethylene and 1-octene binary copolymerization reaction equation, combined with the characteristics of single-center metallocene catalysts that can accurately regulate the chain structure of polyolefins, this study investigates the influence of ethylene, 1-octene, and Ti–CGC feeding sequences on the structure and properties of copolymers. Methylaluminoxane (MAO) was used as the co-catalyst, and the experimental results are summarized in Table 1.

As shown in Table 1, the OCE copolymer exhibits the highest catalytic activity of 1.6 × 10^7^ g polymer/(mol catalyst · h), and the lowest density of 0.889 g/cm^3^ among the three copolymers. EOC copolymer has a slightly lower catalytic activity of 1.4 × 10^7^ g polymer/(mol catalyst · h) with a density of 0.898 g/cm^3^. ECO copolymer shows the lowest catalytic activity of 1.3 × 10^7^ g polymer/(mol catalyst · h) with a density of 0.914 g/cm^3^. The *MFR* of EOC is 0.6 g/10 min, that of ECO is 0.9 g/10 min, and that of OCE is 0.1 g/10 min, indicating that ECO has the best processing performance, while OCE has the worst processing performance.

### 3.1. M_w_ and Molecular Weight Distribution (MWD)

**Figure 1:**
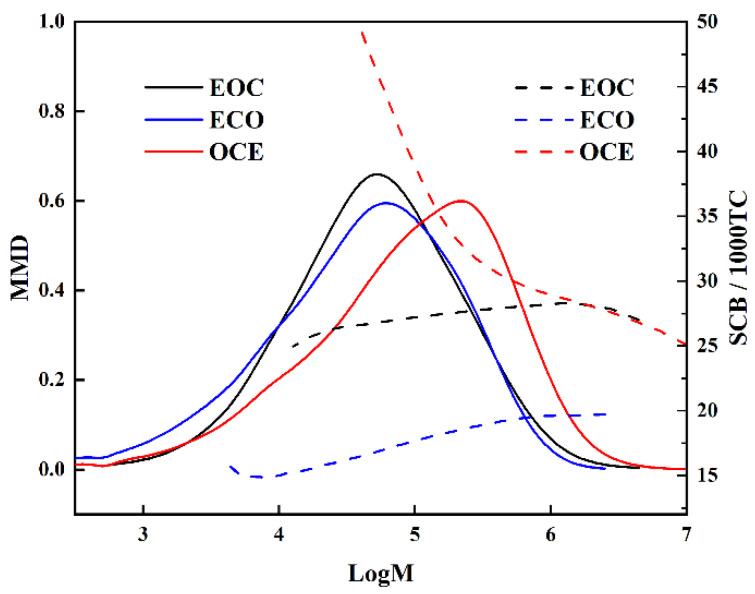
MMD and SCB/1000TC with copolymers molecular weight of EOC, ECO, and OCE.

The number of short-chain branches per 1000 carbons in total (SCB/1000TC) [36] can reflect the content of comonomers. For ethylene/1-octene copolymers synthesis by Ti–CGC, short-chain branches mean hexyl chain branches on the polymer backbone. The larger the value of SCB/1000TC, the more hexyl branches in the copolymer, and the higher the comonomer content in the copolymer. As depicted in Figure 1, the SCB/1000TC of OCE decreases from 55 to 25 as the molecular weight increases, indicating that the 1-octene average content gradually decreases with increasing molecular weight, while in EOC and ECO copolymers, the SCB/1000TC shows slight variation with the molecular weight, indicating that the 1-octene average content remains nearly constant with increasing copolymer molecular weight. The variation of the 1-octene content of EOC, ECO, and OCE copolymers with different molecular weights indicates that controlling the feeding sequence of monomers and catalysts can directly influence the inter-distribution of hexyl chain branches on the polymer backbone.

The *M*_w_ of EOC and ECO are similar, while the OCE has a high *M*_w_. EOC copolymer exhibits the narrowest molecular weight distribution, with a value of 7.4; OCE copolymer has a molecular weight distribution of 11.5, and ECO copolymer has the widest distribution of 14.5. This indicates that the feeding sequences significantly affect the molecular weight distribution of copolymers. The EOC feeding sequence results in the most uniform distribution, and the ECO feeding sequence results in the widest distribution.

During the EOC copolymerization process, ethylene and 1-octene concentrations were simultaneously reduced. This resulted in copolymers with a uniform 1-octene average composition. However, the semi-continuous nature of the process leads to a broader molecular weight distribution than the continuous process. During the initial stages of OCE copolymerization, the 1-octene concentration remained relatively high, while the ethylene concentration gradually increased as the reaction progressed. Consequently, the resulting copolymer exhibited a decrease in 1-octene content. Due to the comonomer effect, the OCE copolymerization had a fast reaction rate. The ethylene concentration remained relatively high in the ECO copolymerization, while the 1-octene concentration increased as the reaction progressed. The rapid growth of ethylene chains in the initial stages of the reaction hindered the incorporation of 1-octene with higher steric hindrance, resulting in a low 1-octene content.

### 3.2. Chemical Composition

**Figure 2:**
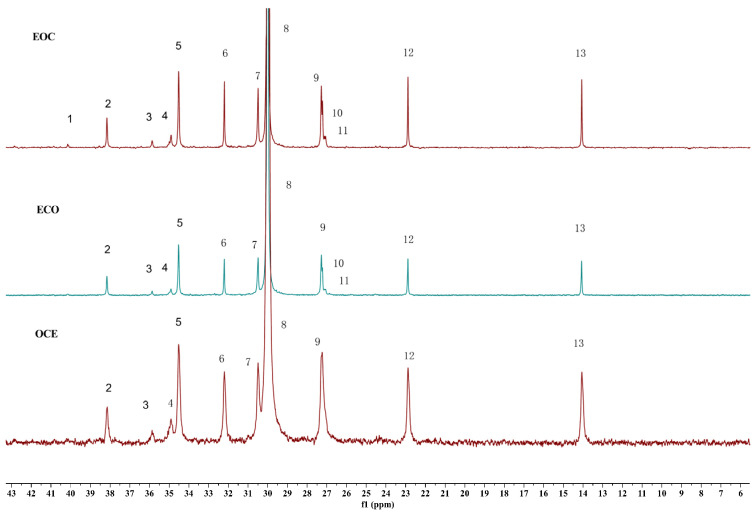
^13^C-NMR of EOC, ECO and OCE.

The ^13^C-NMR spectrums of the copolymers are presented in Figure 2. The nomenclature suggested by Carman and Wilkes [37] is employed. The chemical shift assignments for the peaks are listed in Table 2.

The chemical average composition information of the ethylene/1-octene copolymer was calculated according to Pooter and Smith [34]. The 1-octene content of EOC and OCE copolymers is close, about 21.0 wt%, indicating the feeding sequence of EOC and OCE will not affect the average composition of the copolymer. The 1-octene content of ECO copolymers is the lowest, about 13.2 wt%, indicating the feeding sequence of ECO will reduce the insertion of comonomer. The concentration of 1-octene was low in the early stage of the reaction, and the ethylene chain rapidly grew, inhibiting the incorporation of 1-octene with high steric hindrance, resulting in a decrease in the 1-octene content of the ECO copolymer chain.

The triad distributions, comonomer mole fractions, and copolymerization parameters were calculated using the Randall method [38]. The results are listed in Table 3. For the feeding sequence of the EOC and OCE, *r*_E_ = 28.62 for ethylene, and *r*_o_ = 0.15 for 1-octene for EOC; *r*_E_ = 34.18 for ethylene, and *r*_o_ = 0.18 for 1-octene for OCE, indicating that the copolymerization reaction is beneficial for ethylene incorporation.

### 3.3. Thermal Performance

**Figure 3:**
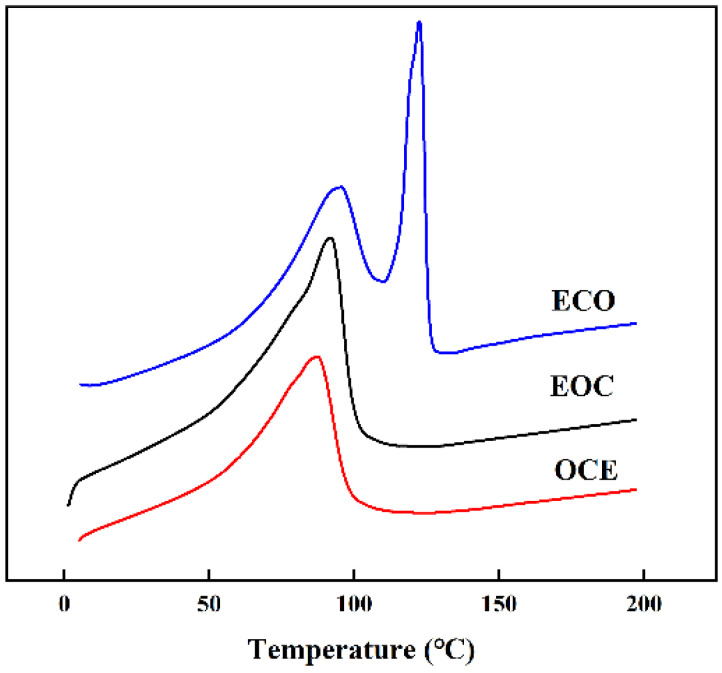
DSC melting curves of ECO, EOC, and OCE.

As depicted in Figure 3, the peak profiles of EOC and OCE copolymers are similar, with a broad peak appearing at 80–90 °C, corresponding to the ethylene/1-octene copolymer peak. The melting point of the OCE copolymer is 87 °C, with a crystallinity of 11.9%. The EOC copolymer exhibits a melting point of 92 °C, with a crystallinity of 14.2%. The comonomer contents of OCE and EOC are close, indicating that the chain microstructure with gradually decreasing comonomer content with increasing molecular weight will lead to lower melting points and crystallinity. The ECO copolymer shows two melting peaks during the melting process. The first peak, at 95 °C, represents the melting of the ethylene/1-octene copolymer, and the second peak, at 123 °C, corresponds to the melting of polyethylene. A small amount of polyethylene is formed in the initial stages of the ECO polymerization reaction. As the 1-octene concentration increases, the polymerization reaction gradually transitions from ethylene homopolymerization to ethylene/1-octene copolymerization.

**Figure 4:**
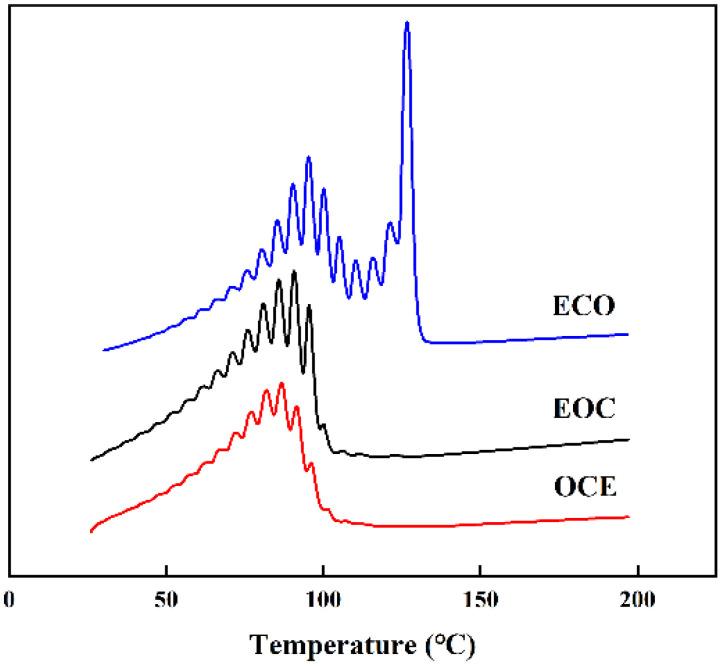
SSA melting curves of ECO, EOC, and OCE.

In Figure 4, the SSA curve profiles of OCE and EOC copolymers are nearly identical. ECO exhibited an additional melting peak in the temperature range of 110–120 °C, with the most substantial melting peak at 121 °C. This indicates that, besides being an ethylene/1-octene copolymer, the ECO copolymer also contains a certain amount of ethylene homopolymer. These results align with the findings from DSC testing.

The methylene sequence length (*MSL*) values and lamellar thickness were calculated according to procedures reported in the literature [39,40]. The analyzed data is listed in Table 4 and Table 5. OCE and EOC copolymers have a similar average lamellar thickness, but OCE has a higher content of low lamellar thickness, indicating that they have similar octene content but different comonomer distributions. Due to high polyethylene crystallization in ECO copolymers, the average lamellar thickness is higher than that of EOC and OCE.

### 3.4. WAXD Analysis

**Figure 5:**
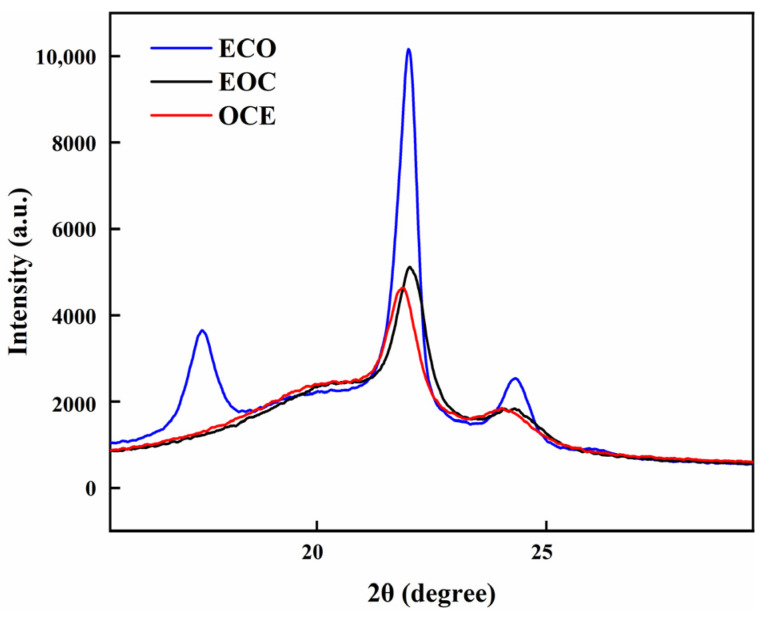
WAXD of ECO, EOC, and OCE copolymers.

Figure 5 shows WAXD diffractograms of the EOC, OCE, and ECO copolymers. The diffraction peaks at 21.8° and 24.2° are the (110) and (200) crystal plane diffraction peaks in the orthogonal crystal cell of polyethylene. Calculate crystallinity is based on diffraction peak intensity according to the following formula; the results are listed in Table 6. The consistency in the variation patterns observed between the WAXD and DSC measurement results indicates the reliability of the crystallinity data obtained from both methods.

(3) $$X_{c}=\frac{I_{110}+I_{200}}{I_{110}+I_{200}+I_{a}}$$

*I*_110_ stands for the area of (110) crystal plane of polyethlene, *I*_200_ stands for the area of (200) crystal plane of polyethylene, and *I*_a_ stands for the area of the amorphous region.

### 3.5. Mechanical Properties

**Figure 6:**
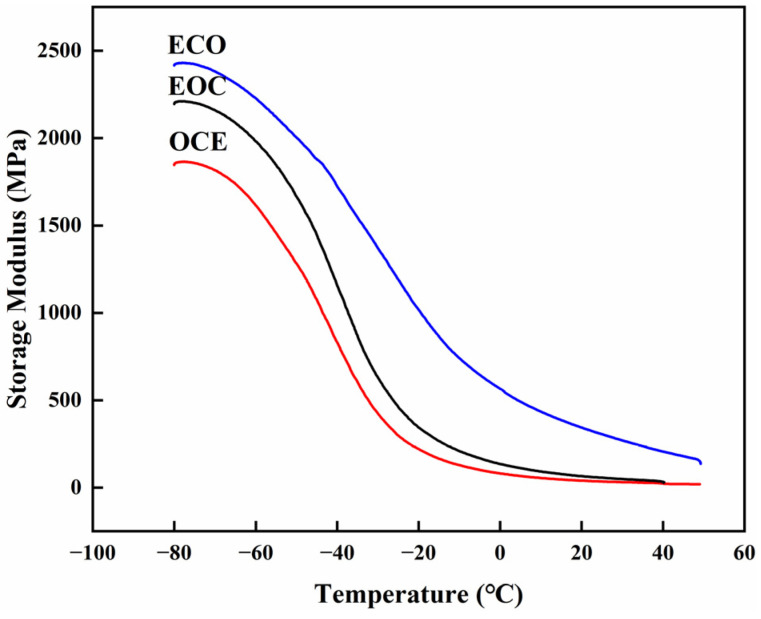
Storage modulus versus temperature curves of ECO, EOC, and OCE.

Figure 6 shows the curves of storage modulus with temperature for three ethylene/1-octene copolymers. The OCE copolymer has the lowest storage modulus, the EOC copolymer has a moderate storage modulus, and the ECO has the highest energy storage modulus, indicating that the OCE copolymer has the best elasticity, while the ECO copolymer has the worst elasticity.

**Figure 7:**
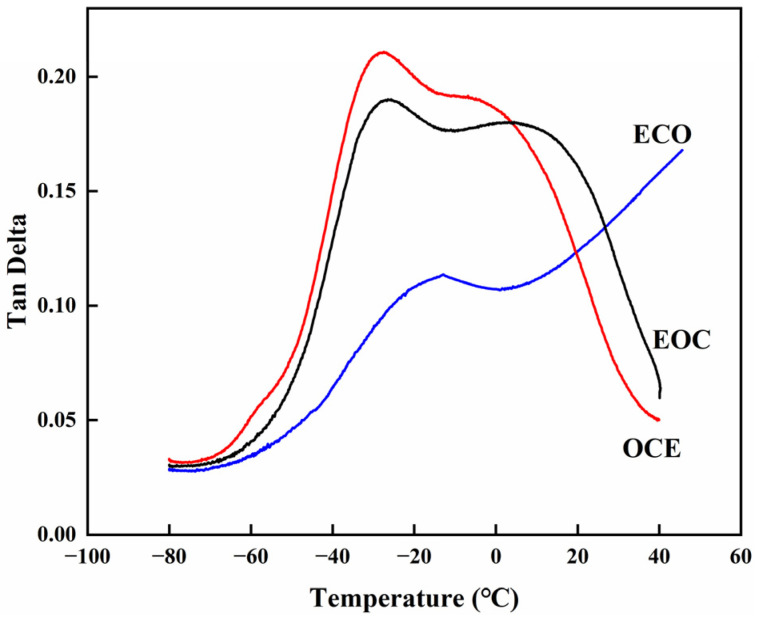
Loss tangent (tan *δ*) versus temperature curves of ECO, EOC, and OCE.

The tan δ curves of the three copolymers are shown in Figure 7. The ECO copolymer and OCE copolymer exhibit an overlapping peak between −75 °C and 50 °C, indicating that the α- and β-transitions of the two copolymers overlap with each other, suggesting a moderate 1-octene content. In addition to a clear peak at −75–10 °C, the ECO copolymer also has a rise in the higher temperature region, indicating that, in addition to the ethylene/1-octene copolymer, the ECO copolymer also contains an amount of ethylene homopolymer.

**Figure 8:**
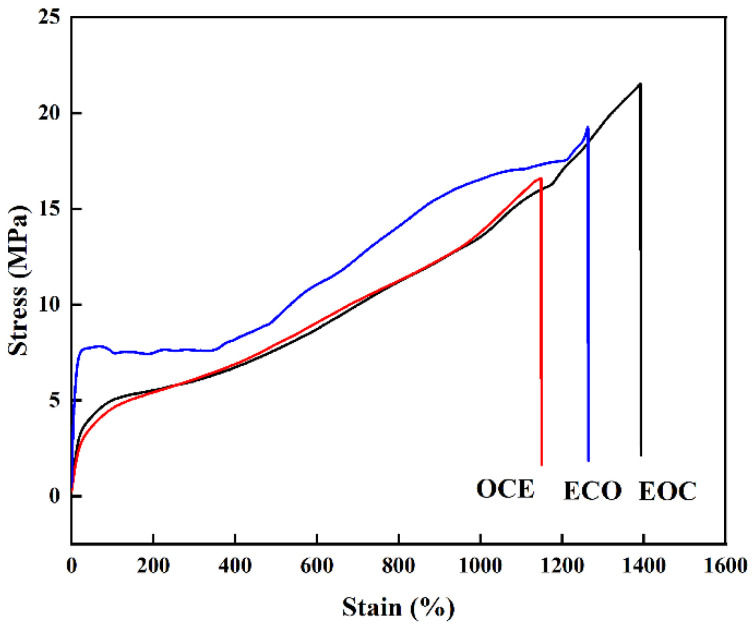
Stress–strain curves of ECO, EOC, and OCE.

As depicted in Figure 8, the stress–strain curves of EOC and OCE are nearly identical. The EOC copolymer exhibits the highest elongation at break, reaching 1430%. The OCE copolymer has the lowest elongation at a break of 1150%. The ECO copolymer has an elongation at a break of 1300%. The elongation at break is related to the content of comonomers in the copolymer and the distribution of comonomers on the backbone, and the EOC feeding sequence leads to a higher elongation at break.

## 4. Conclusions

In this work, we synthesized OCE, EOC, and ECO copolymers with different chain microstructures by controlling the feeding sequence of ethylene, 1-octene, and Ti–CGC in a semi-continuous reaction system. The variation of the 1-octene content of EOC, ECO, and OCE copolymers with different molecular weights indicates that controlling the feeding sequence of monomers and catalysts can directly influence the inter-distribution of hexyl chain branches on the polymer backbone. EOC and OCE different melting points and crystallinity indicate that the chain microstructure with decreasing comonomer content as molecular weight increases will lead to lower melting points and crystallinity. OCE and EOC polymerizations produce a single ethylene/1-octene copolymer, while EOC copolymers, in addition to containing ethylene/1-octene copolymers, also contain a large amount of ethylene homopolymer. This suggests that changing the feeding sequence can control the composition of the copolymer. This method is applied to other ethylene/α-olefin copolymer systems and is essential for developing novel high-performance polyolefin materials.

## Figures and Tables

**Table 1:** Ethylene/1-octene copolymerization ^a^.

Sample	Yield (g)	Act. ^b^	The Content of 1-Octene (wt%) ^c^	*M*_w_ ^d^	*M*_w_	*M*_w_/*M*_n_ ^d^	*T*_m_ (°C)	Density ^e^	*MFR* ^f^
OCE	73	1.6	21.2	2.60	2.60	11.5	87	0.889	0.1
EOC	70	1.4	21.4	1.33	1.33	7.4	92	0.898	0.6
ECO	66	1.3	13.2	1.11	1.11	14.5	95; 123	0.914	0.9

^a^ Reaction Conditions: *T* = 120 °C, *P* = 3 MPa, Ti–CGC = 20 umol, MAO = 6 mL, AlEt_3_ = 3 mL, cyclohexane = 2 L, 1-octene = 300 mL, polymerization time = 15 min. ^b^ Activity is in the unit of 10^7^ g polymer/(mol catalyst · h). ^c^ The content of 1-octene (wt%) in polymer determined by ^13^C-NMR. ^d^ *M*_w_ is in the unit of 10^5^ g/mol. ^e^ Density is in the unit of g/cm^3^, density balance for measurement. ^f^ *MFR* is in the unit of g/10 min.

**Table 2:** Chemical shift assignments in ^13^C-NMR spectra of the EOC, ECO, and OCE.

Peak No.	Carbon Type	Monomer Sequence	Chemical Shift (ppm)		
			ECO	EOC	OCE
1	αα	EOOE		40.15	
2	CH	EOE	38.16	38.16	38.15
3	CH	OOE	35.87	35.87	35.87
4	αδ^+^CH_2_(6)	EEOO + EOO	34.91	34.91	34.91
5	αδ^+^CH_2_(6)	EOEE + EOE	34.52	34.52	34.51
6	CH_2_(3)	EOE + OOE + OOO	32.21	32.21	32.20
7	γδ^+^	OEEE	30.50	30.50	30.49
8	δ^+^δ^++^CH_2_(4)	(EEE)_n_	30.00	30.00	30.00
9	βδ	OEE	27.29	27.29	27.30
10	CH_2_(5)	EOE + OOE + OOO	27.23	27.23	
11	βδ^+^	OOEE	27.09	27.13	
12	CH_2_(2)	EOE + OOE + OOO	22.90	22.89	22.88
13	CH_3_	EOE + OOE + OOO	14.09	14.08	14.06

**Table 3:** Triad sequence distribution (mole fraction) of the EOC, ECO, and OCE copolymers.

Sample	Copolymerization Parameters		Mole Fractions		EEE	EEO	OEO	EOE	OOE	OOO
	*r*_E_	*r*_O_	E	O						
EOC	28.62	0.15	0.96	0.04	0.90	0.06	0	0.03	0.01	0
OCE	34.18	0.18	0.97	0.03	0.91	0.06	0	0.02	0.01	0
ECO	48	--	0.98	0.02	0.94	0.04	0	0.02	0	0

**Table 4:** Lamellar parameters of the EOC, OCE, and ECO copolymers.

	Melting Point/°C	Methylene Sequence Length	Lamella Thickness, *l*c/nm	Share of Fraction/%
EOC	37.5	16.6	2.4	1.7
	42.3	17.9	2.5	2.6
	47.0	19.2	2.6	2.9
	51.8	20.6	2.7	4.4
	56.6	22.2	2.9	4.7
	61.5	24.1	3.1	7.0
	66.4	26.2	3.3	7.0
	71.1	28.4	3.5	8.9
	76.0	31.2	3.7	10.3
	81.0	34.5	4.0	12.4
	85.9	38.3	4.3	13.2
	90.8	42.9	4.7	13.2
	95.5	48.2	5.2	8.8
	99.8	54.3	5.6	1.9
	105.6	64.9	6.5	1.0
OCE	38.5	17.0	2.4	5.1
	43.7	18.3	2.5	2.3
	47.9	19.5	2.6	3.4
	52.4	20.8	2.8	4.3
	57.2	22.4	2.9	5.8
	62.1	24.3	3.1	6.5
	67.1	26.5	3.3	8.5
	72.1	29.0	3.5	9.4
	77.0	31.8	3.8	10.7
	81.9	35.1	4.1	13.3
	86.8	39.0	4.4	12.9
	91.6	43.7	4.8	10.0
	96.2	49.1	5.2	4.5
	100.8	55.9	5.8	1.1
	104.3	62.2	6.3	2.2
ECO	36.2	16.5	2.4	0.3
	43.7	18.3	2.5	1.5
	47.4	19.3	2.6	0.4
	51.7	20.6	2.7	1.5
	56.4	22.1	2.9	1.7
	61.1	23.9	3.1	2.5
	66.1	26.0	3.2	3.2
	71.0	28.4	3.5	3.9
	75.8	31.1	3.7	4.6
	80.5	34.1	4.0	5.9
	85.5	37.9	4.3	7.4
	90.4	42.4	4.7	9.2
	95.4	48.1	5.1	10.1
	100.2	54.9	5.7	7.9
	105.1	63.9	6.4	5.6
	110.4	77.0	7.4	4.6
	115.8	96.6	8.7	5.2
	121.3	128.9	10.7	7.7
	126.6	187.3	13.7	17.0

**Table 5:** Lamellae thickness of EOC, OCE, and ECO.

Sample	*l*_n_/nm	*l*_w_/nm	*l*_w_/*l*_n_
EOC	3.9	15.8	4.1
OCE	3.8	15.1	4.0
ECO	6.9	61.0	8.9

**Table 6:** Crystallinity of OCE, ECO, and ECO copolymers.

Sample	*X*_c_ ^a^	*X*_c_ ^b^
OCE	11.9%	21.4%
EOC	14.2%	23.5%
ECO		37.0%

^a^ *X*_C_ measures by the DSC method. ^b^ *X*_C_ measures by WAXD method.

## Data Availability

The relevant data generated and analyzed in the current study are available from the corresponding author upon reasonable request.
